# Supplementary material for: Thymopentin enhances adenoviral oncolytic therapy by regulating macrophages and CD8+ T cells
Source: Commun Med (Lond). 2026 Mar 16;6:265. doi: 10.1038/s43856-026-01509-6 (PMC13136370; doi:10.1038/s43856-026-01509-6)
Supplement: Supplementary file 2 — Supplemental Information [file 43856_2026_1509_MOESM2_ESM.pdf]

Supplementary Information

Thymopentin enhances adenoviral oncolytic therapy by regulating macrophages and CD8$^+$ T cells

Authors: Kua Liu[2]†, Lingkai Kong[2, 3]†, Huawei Cui[2]†, Peng Wang[2], Jiannan Qiu[2], Qilei Xin[1], Dan Zhou[2], Wencui Liu[1], Fangkun Zhao[2], Junnan Wu[2], Xiaosong Gu[1, 3*], Chunping Jiang[1, 2, 3, 4, 5*], and Junhua Wu[1, 2, 3*]

*Correspondence: wujunhua@nju.edu.cn; chunpingjiang@163.com; nervegu@ntu.edu.cn;

†These authors contributed equally to this work.

Affiliations:

[1] Jinan Microecological Biomedicine Shandong Laboratory, Jinan, 250021, China

[2] State Key Laboratory of Pharmaceutical Biotechnology, School of Life Sciences, National Institute of Healthcare Data Science at Nanjing University, Jiangsu Key Laboratory of Molecular Medicine, Medical School, Nanjing University, Nanjing, 210093, China

[3] Division of Hepatobiliary and Transplantation Surgery, Department of General Surgery Nanjing Drum Tower Hospital, the Affiliated Hospital of Medical School, Nanjing University, Nanjing, 210008, China.

[4] Department of Hepatobiliary and Pancreatic Surgery, The Second Affiliated Hospital of Fujian Medical University, 362000 Quanzhou, Fujian Province, China

[5] Renhuai People's Hospital, 564055 Renhuai, Guizhou Province, China

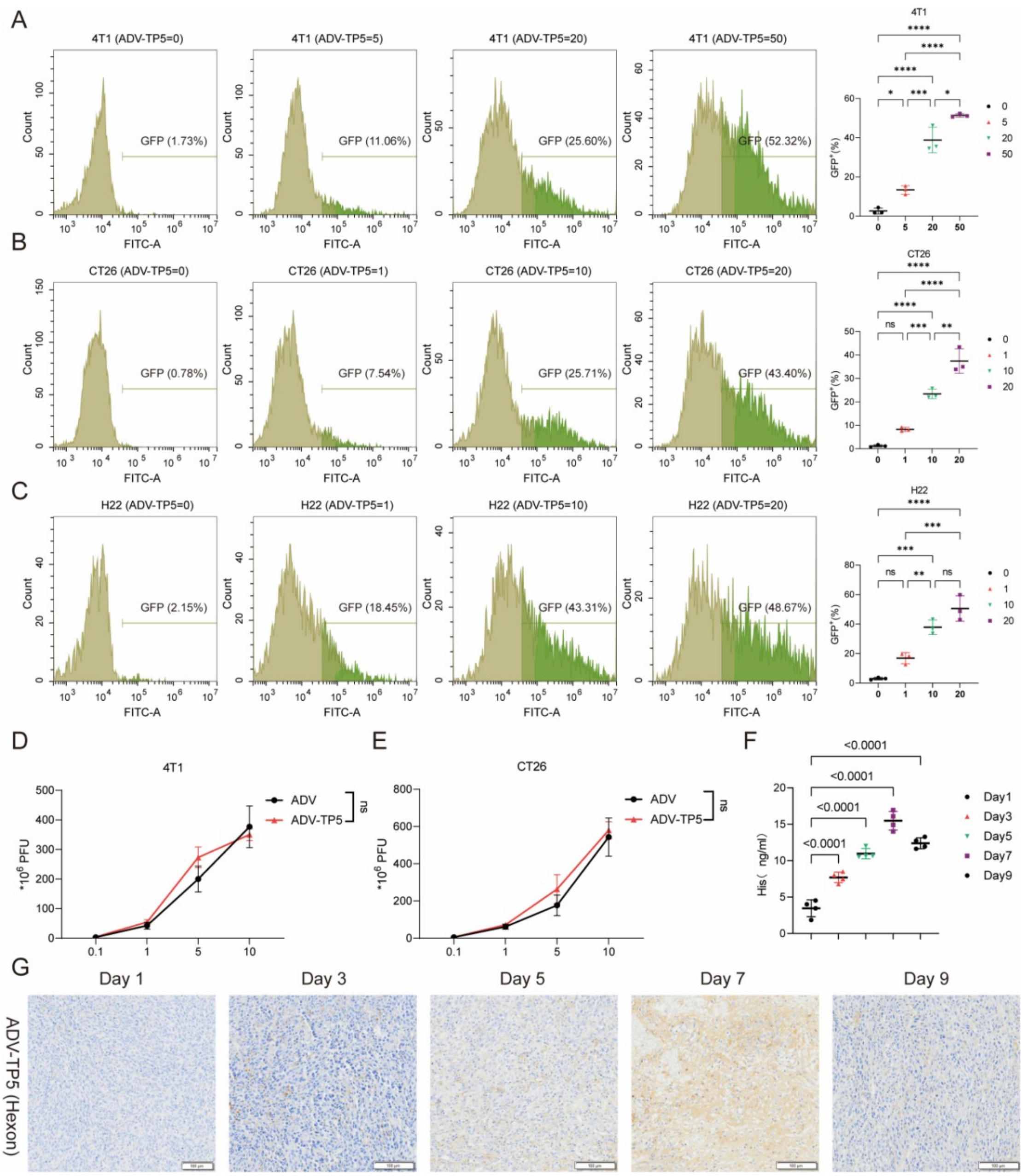

Figure S1. ADV-TP5 infects and replicates in murine tumour cells and expresses TP5. (A-C) 4T1, CT26 and H22 cells were infected with ADV-TP5 at the indicated MOIs, and GFP$^+$ cells were quantified by flow cytometry 48 h after infection. (D, E) 4T1 and CT26 cells were infected with ADV or ADV-TP5 at the indicated MOIs, and viral titers were measured 72 h later using the Adeno-X™ Rapid Titer Kit. (F) 4T1 tumour-bearing mice received intratumoural ADV-TP5, tumours were collected on days 1, 3, 5, 7 and 9, and His levels were determined by ELISA. (G) Hexon expression in tumour tissues was assessed by immunohistochemistry. The data are shown as the means ± SD. ns, no significant difference; *p < 0.05, **p < 0.01, ***p<0.001, ****p < 0.0001.

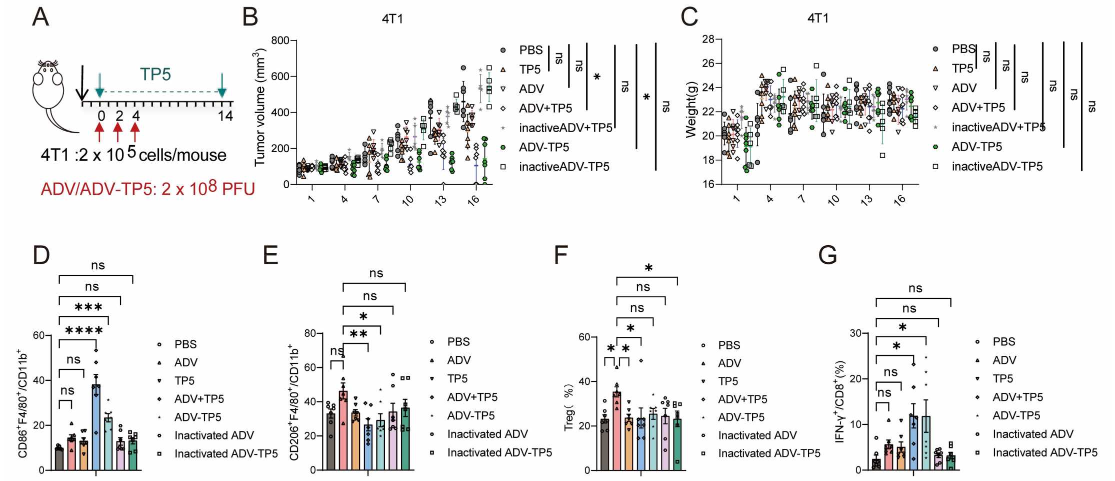

Figure S2. The antitumour efficacy of TP5 combined with ADV depends on replication-competent ADV. (A) Tumour-bearing mice were treated with inactivated or replication-competent ADV or ADV-TP5. (B) Tumour volumes were measured (n = 7). (C) Body weights were monitored (n = 7). (D–G) Tumour tissues were collected and analysed by flow cytometry to assess M1 macrophages, M2 macrophages, Treg cells and IFN-γ$^+$ CD8$^+$ T cells. The data are shown as the means ± SD. ns, no significant difference; *p < 0.05, **p < 0.01, ***p<0.001, ****p < 0.0001.

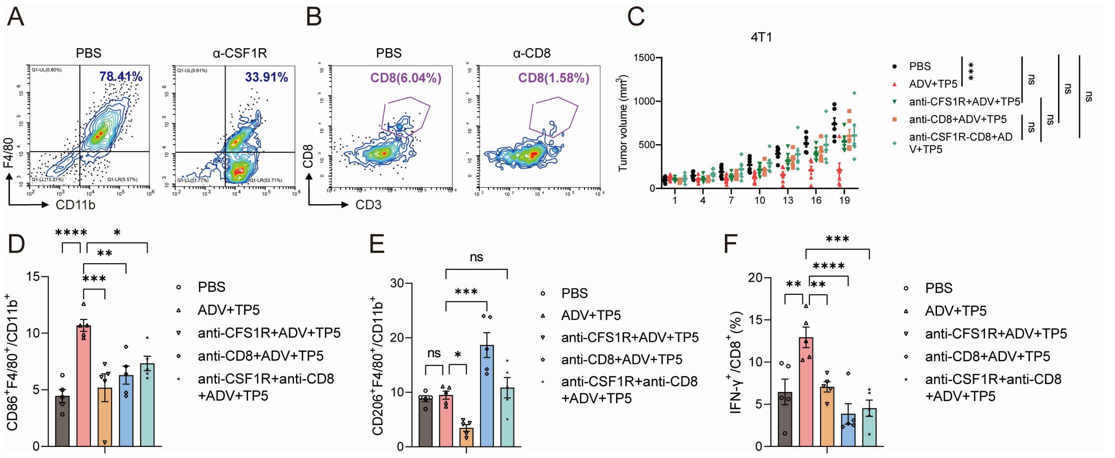

Figure S3. Depletion of CD8$^+$ T cells or macrophages markedly impairs the antitumour efficacy of ADV combined with TP5. (A, B) 4T1 tumour-bearing mice were treated with anti-CSF1R (20 mg/kg) or anti-CD8a (10 mg/kg) neutralizing antibodies, or the combination, followed by ADV plus TP5, and depletion of macrophages (A) and CD8$^+$ T cells (B) was analysed by flow cytometry. (C) Tumour growth curves (n = 6). (D–F) Flow-cytometric analysis of intratumoural M1 macrophages, M2 macrophages and IFN-γ$^+$ CD8$^+$ T cells. The data are shown as the means ± SD. ns, no significant difference; *p < 0.05, **p < 0.01, ***p<0.001, ****p < 0.0001.

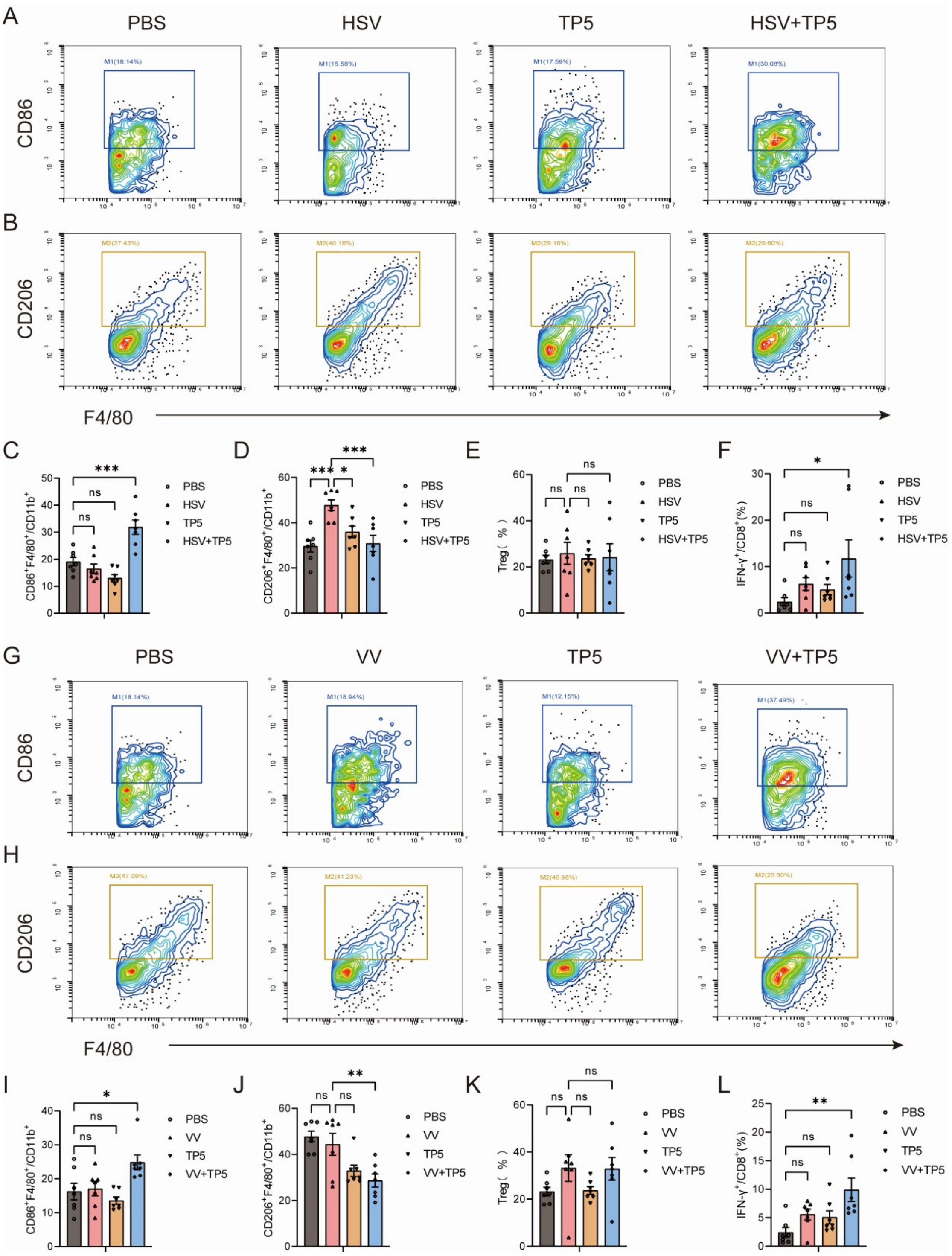

Figure S4. TP5 in combination with HSV and VV promotes infiltration of M1 macrophages and cytotoxic CD8+ T cells and enhances the antitumour efficacy of HSV and VV. (A, B) 4T1 tumour-bearing mice were treated with HSV, TP5 or the combination (HSV + TP5). 14 days after treatment, tumours were harvested and tumour-infiltrating immune cells were analysed by flow cytometry. The frequencies of intratumoural M1 macrophages ($CD86^+$ TAMs) (C), M2 macrophages ($CD206^+$ TAMs) (D), Treg cells (E) and IFN-γ^+CD8^+ T cells (F) (n = 6 mice per group) were quantified. (G, H) 4T1 tumour-bearing mice were treated with VV, TP5 or the combination (VV + TP5). 14 days after treatment, tumours were harvested and tumour-infiltrating immune cells were analysed by flow cytometry. The frequencies of intratumoural M1 macrophages ($CD86^+$ TAMs) (I), M2 macrophages ($CD206^+$ TAMs) (J), Treg cells (K) and IFN-γ^+CD8^+ T cells (L) (n = 6 mice per group) were quantified. The data are shown as the means ± SD. ns, no significant difference; *p <0.05, **p <0.01, ***p <0.001.

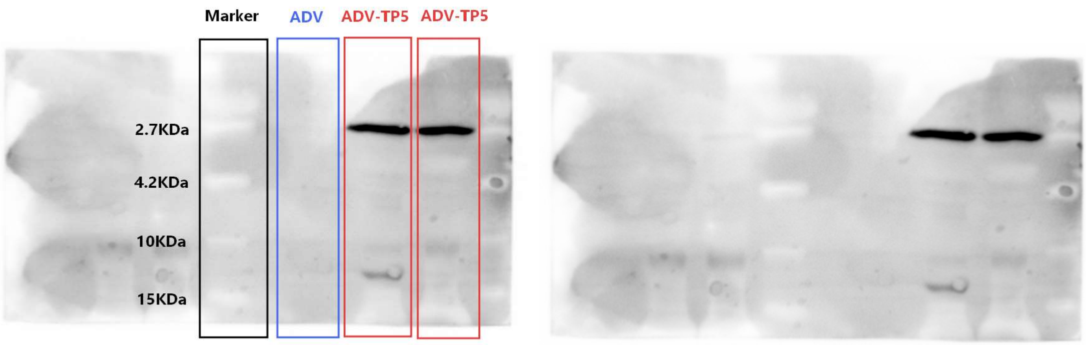

Figure S5. Full, uncropped images of western blots related to Fig. 4B. Blot for TP5-His tag using anti-His antibody.
