# Supplementary material for: Thymopentin enhances adenoviral oncolytic therapy by regulating macrophages and CD8+ T cells
Source: Commun Med (Lond). 2026 Mar 16;6:265. doi: 10.1038/s43856-026-01509-6 (PMC13136370; doi:10.1038/s43856-026-01509-6)
Supplement: Supplementary file 3 — Description of Additional Supplementary Files [file 43856_2026_1509_MOESM3_ESM.docx]

**Description of Additional Supplementary Files**

File name: Supplementary Data 1-7

Description: Source Data for main figures

File name: Supplementary Data 8-11

Description: Source Data for supplementary figures
